# Taste receptor type 1 member 3 in osteoclasts regulates osteoclastogenesis *via* detection of glucose

**DOI:** 10.1016/j.jbc.2025.108273

**Published:** 2025-02-06

**Authors:** Anna Yoshimura, Takuma Matsubara, Nao Kodama, Yoshimitsu Kakuta, Kazuma Yasuda, Ryusuke Yoshida, Osamu Kaminuma, Shuhei Hosomi, Hiroji Shinkawa, Quan Yuan, Tatsuo Kawamoto, Shoichiro Kokabu

**Affiliations:** 1 Division of Molecular Signaling and Biochemistry, Kyushu Dental University, Kitakyushu, Fukuoka, Japan; 2 Division of Orofacial Functions and Orthodontics, Kyushu Dental University, Kitakyushu, Fukuoka, Japan; 3 Laboratory of Structural Biology, Graduate School of Systems Life Sciences, Kyushu University, Fukuoka, Japan; 4 Department of Oral Physiology, Graduate School of Graduate School of Medicine, Dentistry and Pharmaceutical Sciences, Okayama University, Okayama, Japan; 5 Department of Disease Model, Research Institute of Radiation Biology and Medicine, Hiroshima University, Hiroshima, Japan; 6 Department of Gastroenterology, Graduate School of Medicine, Osaka Metropolitan University, Osaka, Japan; 7 Department of Hepatobiliary-Pancreatic Surgery, Graduate School of Medicine, Osaka Metropolitan University, Osaka, Japan; 8 State Key Laboratory of Oral Diseases & National Center for Stomatology & National Clinical Research Center for Oral Diseases, West China Hospital of Stomatology, Sichuan University, Chengdu, China

**Keywords:** glucose, osteoclasts, p38, TAS1R3, taste receptor

## Abstract

The taste system extends beyond the oral cavity, with various taste receptors found in extraoral organs. Mice deficient in the taste receptor type 1 (TAS1R) family member, TAS1R3, and fed a high-fat, high-sugar diet showed high bone mass without altering food consumption. However, the underlying mechanisms, including the cell types responsible for TAS1R3 expression, remain unclear. Here, we demonstrate the expression and function of TAS1R3 in osteoclasts, which are responsible for bone resorption. The expression of *Tas1r3*, but not *Tas1r1* or *Tas1r2*, is evoked during osteoclast differentiation. Osteoclastogenesis-related genes were downregulated in TAS1R3-deficient mice, whereas the opposite phenotypes were elicited by TAS1R3 overexpression. Contrary to the common heterodimerization with TAS1R1 or TAS1R2, TAS1R3 formed a homodimer that functioned to detect glucose, enhance p38 phosphorylation, and induce osteoclastogenesis. These results provide novel insights into the role of TAS1R3 in bone metabolism and suggest that TAS1R3 may be a viable target for therapeutic agents in bone metabolic diseases.

Sensory mechanisms crucial for living animals have diversified extensively through evolution to detect changes in the external environment. Taste sensation is a sensory mechanism dedicated to the detection of chemical compounds within the oral cavity. There are five basic tastes (sweet, bitter, salty, umami, and sour) in humans and rodents, each of which serves to identify beneficial or potentially harmful compounds in food. Notably, the taste system extends beyond the oral cavity, with various taste receptors found in extraoral organs, such as the intestine, brain, respiratory tract, and pancreas (1). These extraoral taste receptors contribute to coordinated organ functions by detecting chemical compounds in their respective environments.

The taste receptor type 1 (TAS1R) family members, TAS1R1, TAS1R2, and TAS1R3, are G-protein–coupled receptors (GPCRs) that form functional taste receptors *via* heterodimerization (2). When TAS1R3 heterodimerizes with TAS1R1, this complex functions as an umami receptor that detects amino acids and nucleic acids. In contrast, TAS1R2–TAS1R3 complex binds to multiple sweeteners, including sugars and artificial sweeteners, and functions as a sweet taste receptor (3, 4, 5, 6). Both sweet and umami receptors interact with heterotrimeric G proteins composed of α-gustducin, Gβ3, and Gγ13. Upon dissociation of the G protein subunit, the Gβγ subunit interacts with phospholipase C-β2, which cleaves phosphatidylinositol 4,5-bisphosphate into inositol 1,4,5-triphosphate (IP_3_). IP_3_ stimulates Ca^2+^ release from the endoplasmic reticulum by activating type III IP3 receptors (IP_3_-R3). Increased intracellular Ca^2+^ activates transient receptor potential cation channel subfamily M member 5, leading to membrane depolarization and generation of action potentials. ATP is then released from taste cells and stimulates afferent nerve fibers (2). Recent studies have shown that the TAS1R family is also found in tissues other than the oral mucosa, where it acts as a nutrient sensor to monitor energy and nutritional status (7).

Bone homeostasis is maintained by the remodeling of osteoclasts and osteoblasts. Osteoclasts absorb the bone matrix during the first step of bone remodeling. Osteoblasts then fill the resorbed area to form new bone. The balance between osteoclast-mediated bone resorption and osteoblast formation is critical for bone homeostasis. When this balance is disrupted and osteoclast function becomes dominant, bone metabolic diseases, such as osteoporosis, occur (8, 9). Osteoclasts are tartrate-resistant acid phosphatase (TRAP)–positive multinuclear cells, which are the only cells that resorb the bone and differentiate from hematopoietic stem cells in response to receptor activator of nuclear factor-kappa B ligand (RANKL) secreted by osteoblasts (10). During osteoclast differentiation, RANKL upregulates NF-κB and mitogen-activated protein kinases such as p38 and extracellular signal–regulated kinase (10, 11, 12, 13). These molecules individually transmit signals that ultimately activate nuclear factor of activated T cells 1 (NFATc1), the master regulator of osteoclast differentiation, and induce osteoclast differentiation and bone resorption.

The severity of obesity and diabetes correlates with the degree of bone destruction caused by periodontitis (14). The risk of fractures increases in obese and diabetic patients (15). Osteoclasts are rich in mitochondria for aerobic respiration and have a high carbohydrate (energy and sugar) demand because they move around the bone surface and actively resorb the bone (16, 17). However, the mechanism of carbohydrate sensing in osteoclasts and the effect of hyperglycemia on osteoclast behavior remain unknown.

Members of the TAS1R family may also play important roles in the bone tissue. In TAS1R3 null mice fed a high-fat, high-sugar (HFS) diet, the femur exhibited increased bone mineral density and thickness in both the trabecular and cortical bones (18). These phenotypes were confirmed by analyzing the loss of function in TAS1R3 mutant mice; the cortical bone thickness in mutant mice was increased by 13% compared with WT mice (19). In addition, TAS1R3 mutant mice exhibited a more than 60% reduction in the serum levels of the bone resorption marker collagen type 1 C-telopeptide (CTx), and increased TAS1R3 expression was strongly correlated with elevated expression of the mature osteoclast marker, cathepsin K (Ctsk). However, the number of osteoblasts and serum levels of the bone formation marker procollagen type 1 N-terminal propeptide in TAS1R3 mutant mice did not differ from those in WT mice (19), suggesting that TAS1R3 regulates osteoclast function. However, cellular and molecular mechanisms underlying these phenotypes remain unclear.

In this study, we demonstrated that TAS1R3 is expressed in osteoclasts and that TAS1R3 expression increases with osteoclast differentiation. The capacity for osteoclast differentiation is reduced in TAS1R3-knockout mice. In contrast, TAS1R3-mediated glucose signaling promotes osteoclast differentiation. Finally, we found that the glucose–TAS1R3 axis promotes osteoclastogenesis by increasing the phosphorylation of p38.

## Result

### TAS1R3 is expressed in osteoclasts and regulates osteoclastogenesis

**Figure 1:**
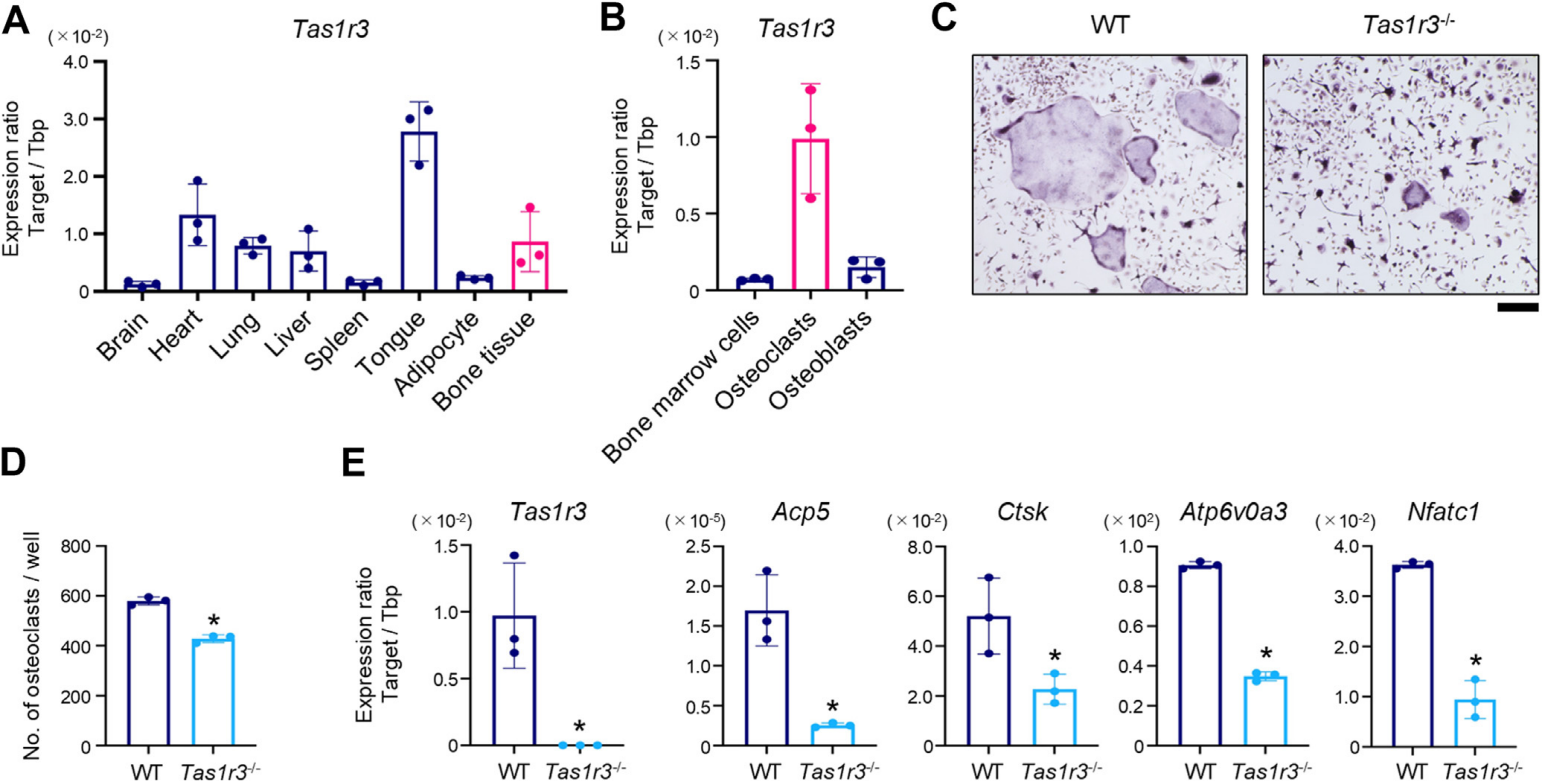
**TAS1R3 is expressed in osteoclasts and regulates osteoclastogenesis.***A* and *B*, mRNA levels of *Tas1r3* in various tissues from 4-week-old mice (*A*), their bone marrow, osteoclasts differentiated from the bone marrow from 4-week-old mice, and osteoblasts obtained from newborn mouse calvaria (*B*) were determined by quantitative PCR. *C* and *D*, osteoclasts were differentiated from bone marrow cells collected from WT or *Tas1r3* conventional knockout (*Tas1r3*^−/−^) mice. Osteoclastogenesis was stimulated by adding both 30 ng/ml M-CSF and 100 ng/ml sRANKL. Osteoclasts were visualized by TRAP staining (*C*), and the number of TRAP-positive cells with three or more nuclei was counted (*D*). Representative images of three independent experiments with similar results are shown. Scale bar represents 200 μm (*C*). *E*, mRNA levels of *Tas1r3*, *Acp5*, *Ctsk*, *Atp6v0a3*, and *Nfatc1* were determined by quantitative PCR on day 7. *A*, *B*, *D*, and *E*, the bar graphs (mean ± SD) with dot plots presenting each sample from three or four independent experiments are shown (∗*p* < 0.05). M-CSF, macrophage colony-stimulating factor; RANKL, receptor activator of nuclear factor-kappa B ligand; TAS1R3, taste receptor type 1 member 3; TRAP, tartrate-resistant acid phosphatase.

First, we examined the mRNA levels of *Tas1r3* in various tissues and cells (Fig. 1, *A* and *B*). *Tas1r3* is strongly expressed in osteoclasts. In contrast, the expression level of *Tas1r3* was low in osteoblasts (Fig. 1*B*). To determine the physiological function of TAS1R3 expressed by osteoclasts, we used the bone marrow cells (bone marrow–derived macrophages [BMMs]) isolated from 4- to 6-week-old *Tas1r3*^−/−^ mice. Under TAS1R3-knockout conditions, the number of TRAP-positive osteoclasts (Fig. 1, *C* and *D*) was significantly reduced. Essentially the same phenotype was obtained by using BMMs from 13-week-old mice (Fig. S1). The expression of osteoclast differential-marker genes, such as *Acp5*, *Ctsk*, *Atp6v0a3*, and *Nfatc1* (Fig. 1*E*), was significantly reduced. These results indicated that TAS1R3 expressed in osteoclasts regulates osteoclast differentiation.

### TAS1R3 detects glucose or cyclamate without TAS1R1 or TAS1R2

**Figure 2:**
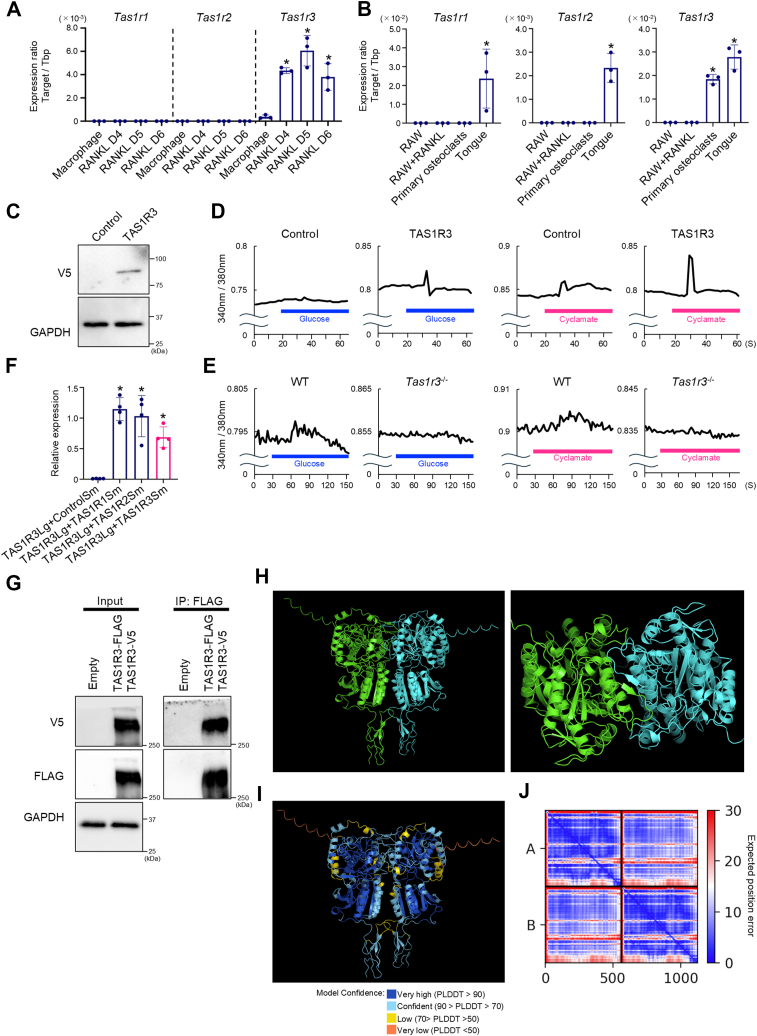
**TAS1R3 detects glucose or cyclamate without TAS1R1 or TAS1R2.***A*, bone marrow cells were collected from WT mice. mRNA levels of *Tas1r1*, *Tas1r2*, and *Tas1r3* were determined by quantitative PCR on day 4 (D4), day 5 (D5), and day 6 (D6) after 100 ng/ml sRANKL stimulation. The bar graphs (mean ± SD) with dot plots presenting each sample from each mouse are shown (∗*p* < 0.05, compared with macrophage). *B*, mRNA levels of *Tas1r1*, *Tas1r2*, and *Tas1r3* in RAW 264.7 cells with or without 100 ng/ml sRANKL, primary osteoclasts, or tongue were determined by quantitative PCR. sRANKL treatment was performed for 3 days. The bar graphs (mean ± SD) with dot plots presenting each sample from the three independent experiments are shown (∗*p* < 0.05, compared with RAW + RANKL). *C*, RAW 264.7 cells stably expressing empty vector (Control) or TAS1R3-V5 (TAS1R3) were generated. The protein levels of V5-tagged TAS1R3 were determined by Western blotting. The entire gel images are placed in Fig. S2, *A* and *B*. *D* and *E*, the intracellular calcium dynamics was measured *via* calcium imaging after adding glucose or cyclamate to Control and TAS1R3 cells (*D*) or BMMs from WT and *Tas1r3*^−/−^ mice (*E*). *F*, HEK293 cells were cotransfected with TAS1R3-LgBiT (TAS1R3Lg) along with Control-SmBiT (ControlSm), TAS1R1-SmBiT (TAS1R1Sm), TAS1R2-SmBiT (TAS1R2Sm), or TAS1R3-SmBiT (TAS1R3Sm). Two days after transfection, luciferase activities were measured. The bar graphs (mean ± SD) with dot plots presenting each sample from four independent experiments are shown (∗*p* < 0.05, compared with TAS1R3Lg + ControlSm). *G*, TAS1R3-V5 were transfected in HEK293 cells along with TAS1R3-FLAG and immunoprecipitated with DYKDDDDK Fab-Trap. Protein levels were determined by Western blotting. The entire gel images are placed in Fig. S2, *C*–*E*. *C*, *D*, *E*, and *G*, representative images of three independent experiments with similar results are shown. *H*, predicted mouse TAS1R3 (1–356) homodimer structure. Each molecule constituting the homodimer is depicted in *green* and *teal*, respectively. *I*, colors represent the predicted local distance difference test (PLDDT) scores. *J*, predicted aligned error (PAE) diagram for the homodimer of TAS1R3 (residues 1–236), consisting of molecules A and B. BMM, bone marrow–derived macrophage; HEK293, human embryonic kidney 293 cell line; RANKL, receptor activator of nuclear factor-kappa B ligand; TAS1R, taste receptor type 1.

Generally, TAS1R3 forms a heterodimer with TAS1R1 or TAS1R2 to sense sugars, amino acids, and nucleic acids as taste receptors in the taste buds. Our next question was whether TAS1R1 and TAS1R2 are required for osteoclastogenesis in addition to TAS1R3 and what ligand activates TAS1R3 during osteoclastogenesis. The expression of both *Tas1r1* and *Tas1r2* was not detected in BMMs, osteoclast precursor cells, or osteoclasts derived from mouse bone marrow, although *Tas1r3* expression increased during osteoclast differentiation (Fig. 2*A*). Interestingly, the expression of *Tas1r3* was reduced on day 6. It has been demonstrated that osteoclast differentiation from BMMs occurs up to 5 days upon RANKL stimulation at least *in vitro*. Then the osteoclasts go into the maturation stage (20, 21). Therefore, our data showing the diminishment of *Tas1r3* expression on day 6 suggest that the participation of TAS1R3 is larger in osteoclast differentiation than in its maturation. RAW 264.7 cells, which have the potential to differentiate into osteoclasts under appropriate stimuli, did not express *Tas1r1*, *Tas1r2*, or *Tas1r3* (Fig. 2*B*). Taking advantage of this feature of RAW 264.7 cells, we generated cells stably overexpressing TAS1R3 (TAS1R3 cells) for further experiments (Fig. 2*C*). When these cells were stimulated with the candidate ligand such as glucose or cyclamate, the intracellular calcium concentration increased significantly in TAS1R3 cells, whereas such increases in intracellular calcium concentration were not observed in control cells (Fig. 2*D*). We next investigated the physiological function of TAS1R3 as a glucose detector. Stimulation with glucose or cyclamate did not alter the intracellular calcium concentration in BMMs isolated from *Tas1r3*^−/−^ mice, whereas WT BMMs responded to these stimuli (Fig. 2*E*). We then conducted binding experiments using HiBiT technology (Fig. 2*F*) and immunoprecipitation (IP) (Fig. 2*G*). The results of the experiments using the HiBiT technology showed that the luciferase activity between TAS1R3s increased to the same level as that between TAS1R3 and TAS1R1 or TAS1R2 (Fig. 2*F*). Furthermore, IP experiments revealed a band of TAS1R3 tagged with V5 upon IP with FLAG (Fig. 2*G*). These results suggested that TAS1R3 forms a homodimer. Therefore, we performed computer simulations using the amino acid sequence of TAS1R3 to predict the homodimeric structure of TAS1R3. The TAS1R3 homodimer was assumed to have the structure shown in Figure 2*H*. The accuracy of these positional relationships was examined, and most were blue or light blue, indicating high reliability in predicting the position of each molecule (Fig. 2*I*). Positional errors between the residues were also low, indicating that the prediction of the homodimeric structure of TAS1R3 was highly accurate (Fig. 2*J*). These data suggested that TAS1R3 forms homodimers and transmits cell signals in response to glucose or cyclamate in the absence of TAS1R1 or TAS1R2.

### TAS1R3, particularly in combination with glucose or cyclamate, enhances osteoclastogenesis

**Figure 3:**
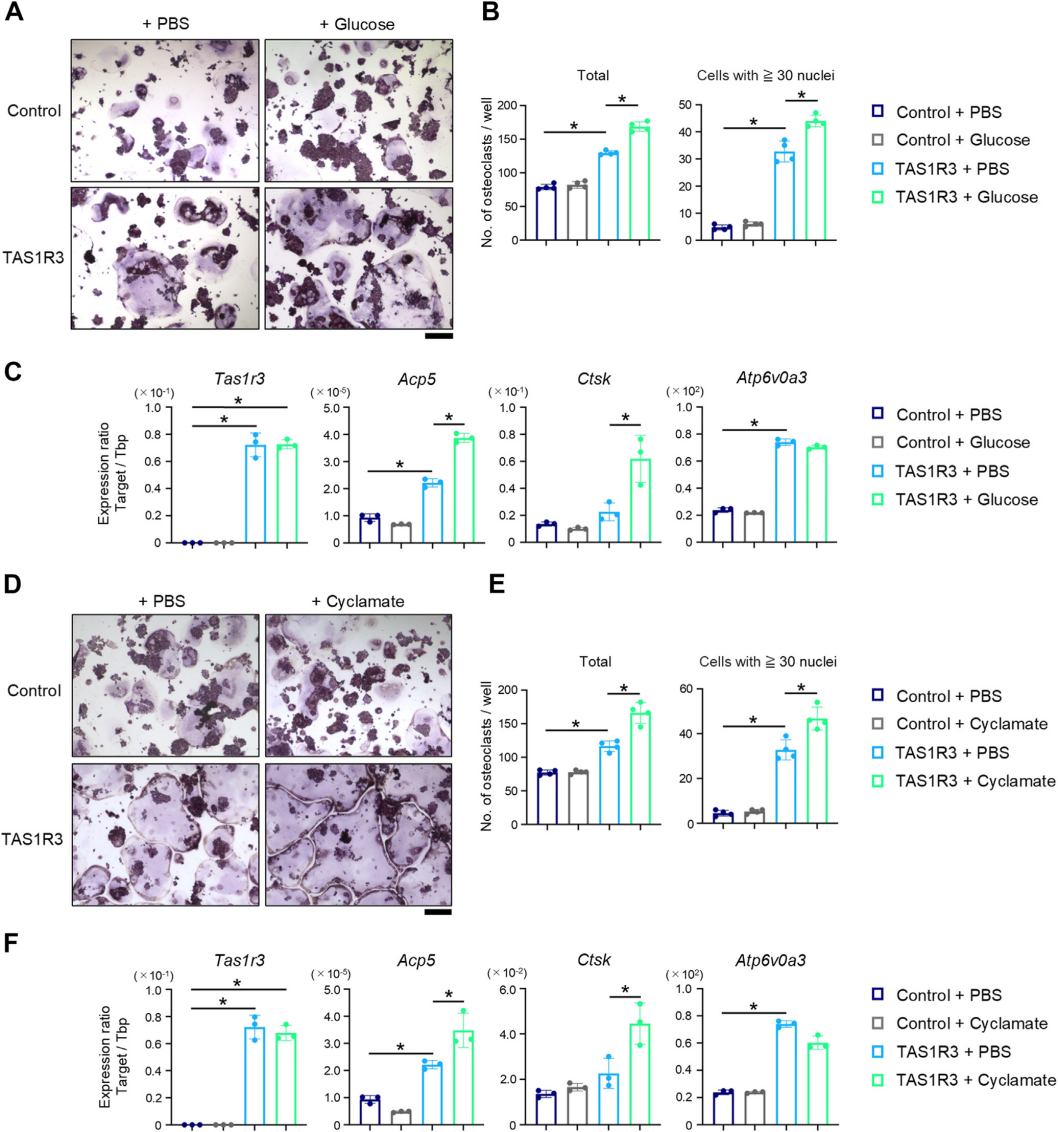
**Glucose or cyclamate promotes osteoclastogenesis *via* TAS1R3 in RAW 264.7 cells.** RAW 264.7 cells stably expressing an empty vector (Control) or TAS1R3-V5 (TAS1R3) were generated. To induce osteoclast differentiation, control or TAS1R3 cells were treated with 50 ng/ml sRANKL. Cells were also treated with 5 mM glucose (*A*–*C*) or 1 μM cyclamate (*D*–*F*) for 4 days. *A* and *D*, representative images of osteoclasts visualized by TRAP staining from three independent experiments with similar results are shown. Scale bar represents 200 μm. *B* and *E*, the number of TRAP-positive cells with three or more nuclei (total) and 30 or more nuclei (cells with ≥30 nuclei) were counted. *C* and *F*, the expression levels of *Tas1r3*, *Acp5*, *Ctsk*, *or Atp6v0a3* were determined by quantitative PCR. *B*, *C*, *E*, and *F*, the bar graphs (mean ± SD) with dot plots presenting each sample from three or four independent experiments are shown (∗*p* < 0.05). RANKL, receptor activator of nuclear factor-kappa B ligand; TAS1R, taste receptor type 1; TRAP, tartrate-resistant acid phosphatase.

**Figure 4:**
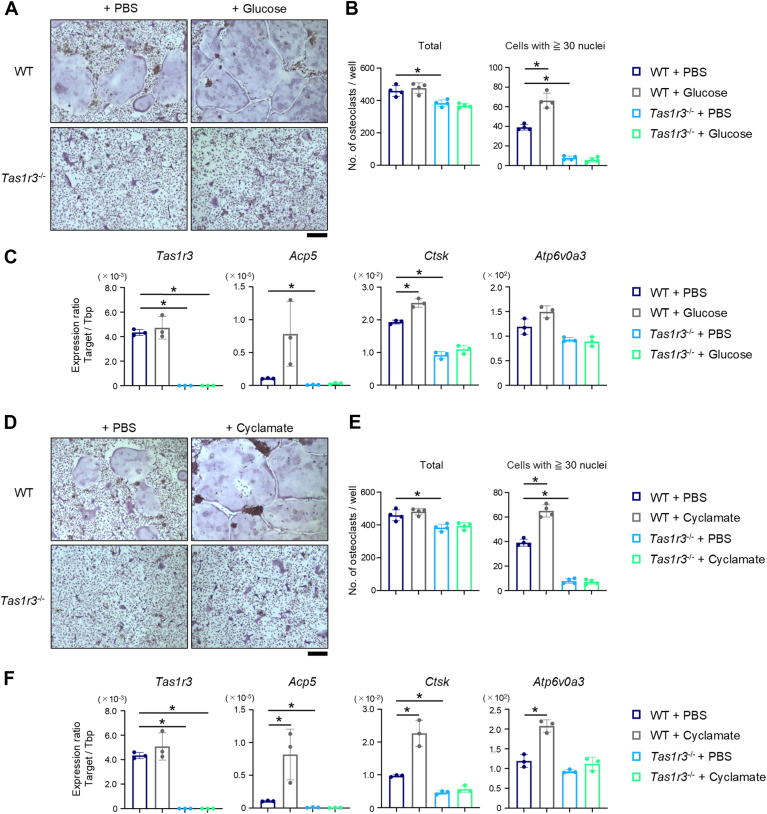
**Glucose or cyclamate promotes osteoclastogenesis *via* TAS1R3 in murine bone marrow cells.** Bone marrow cells from WT or *Tas1r3* conventional knockout (*Tas1r3*^−/−^) mice were cultured in the presence of 30 ng/ml M-CSF and 90 ng/ml RANKL, with or without 2 mM glucose (*A*–*C*) or 1 μM cyclamate (*D*–*F*). *A* and *D*, representative images of osteoclasts visualized by TRAP staining from three independent experiments with similar results are shown. Scale bar represents 200 μm. *B* and *E*, the number of TRAP-positive cells with three or more nuclei (total) and 30 or more nuclei (cells with ≥30 nuclei) were counted. *C* and *F*, the expression levels of *Tas1r3*, *Acp5*, *Ctsk*, *or Atp6v0a3* were determined by quantitative PCR. *B*, *C*, *E*, and *F*, the bar graphs (mean ± SD) with dot plots presenting each sample from three or four independent experiments are shown (∗*p* < 0.05). M-CSF, macrophage colony-stimulating factor; RANKL, receptor activator of nuclear factor-kappa B ligand; TAS1R, taste receptor type 1; TRAP, tartrate-resistant acid phosphatase.

To investigate the effect of excessive TAS1R3 signaling on osteoclastogenesis, TAS1R3 cells were stimulated with RANKL. The number of TRAP-positive osteoclasts, particularly those with 30 or more nuclei, was higher in TAS1R3 cells than in control cells (Fig. 3, *A* and *B*). In addition, the expression of osteoclast differentiation markers, such as *Acp5*, *Ctsk*, and *Atp6v0a3*, was higher in TAS1R3 cells than in control cells (Fig. 3*C*). These results suggest that TAS1R3 overexpression promotes osteoclastogenesis. The effect of TAS1R3 on osteoclast differentiation was further tested using glucose as the TAS1R3 ligand. Treatment of TAS1R3 cells with RANKL and glucose resulted in an increase in the number of TRAP-positive osteoclasts (Fig. 3, *A* and *B*) and enhanced the expression of osteoclast differentiation marker genes (Fig. 3*C*). We confirmed this phenomenon using the artificial sweetener cyclamate. Cyclamate also increased the number of TRAP-positive osteoclasts, particularly those with 30 or more nuclei (Fig. 3, *D* and *E*) and the expression of osteoclast differentiation marker genes (Fig. 3*F*). Next, we induced osteoclastogenesis of BMMs isolated from WT or *Tas1r3*^−/−^ mice. Supplementation of glucose (Fig. 4, *A*–*C*) or cyclamate (Fig. 4, *D*–*F*) increased the number of TRAP-positive cells and osteoclast differentiation marker genes in the cells from WT mice. In addition to the downregulation of osteoclastogenesis and related gene expression, unresponsiveness to glucose or cyclamate was observed in BMMs from *Tas1r3*^−/−^ mice. These data suggested that glucose–TAS1R3 axis is important for physiological osteoclastogenesis.

### Glucose–TAS1R3 axis promotes osteoclast differentiation by increasing phosphorylated p38

**Figure 5:**
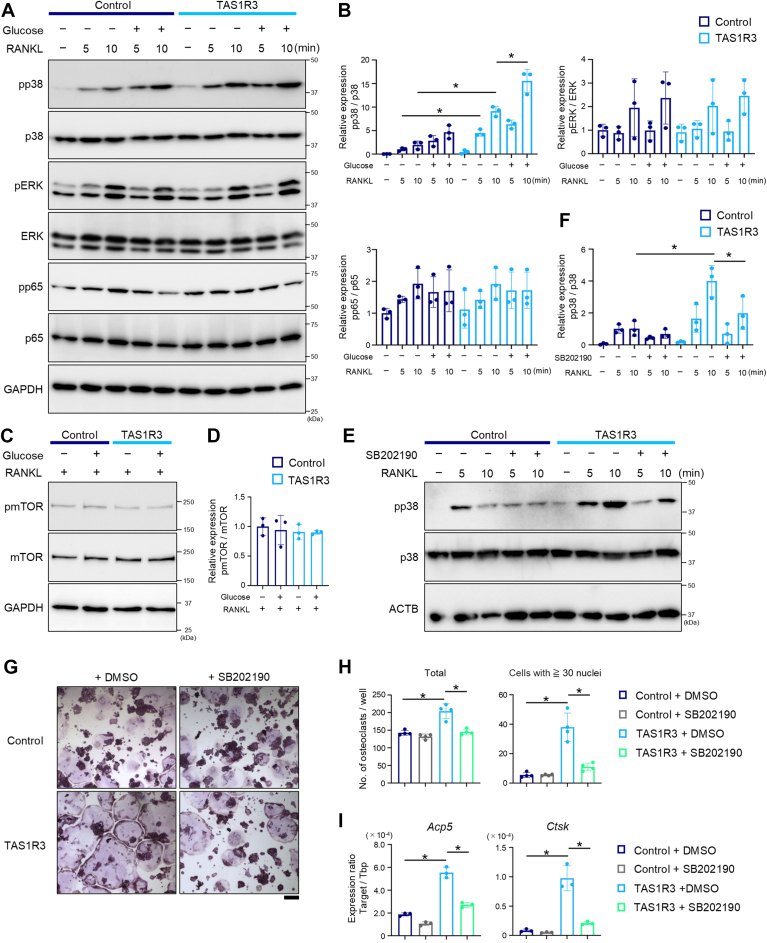
**Glucose–TAS1R3 axis promotes osteoclast differentiation by changing phosphorylated status of p38 in RAW 264.7 cells.***A* and *B*, RAW 264.7 cells stably expressing an empty vector (Control) or TAS1R3-V5 (TAS1R3) were treated with 100 ng/ml sRANKL and with 5 mM glucose for 5 and 10 min. The whole cell lysates were subjected to Western blotting to determine indicated proteins. *A*, the representative blotting images from three independent experiments with similar results are shown. The entire gel and all duplicate images are placed in Figs. S3 and S4. *B*, upon quantification of each blot band, the phospho-protein/whole-protein ratio of indicated proteins was calculated. *C* and *D*, control or TAS1R3 cells were treated with 100 ng/ml sRANKL and 5 mM glucose for 24 h. *E* and *F*, cells were pretreated with SB202190 (0.1 μM) for 1 h. After washing with PBS, cells were treated with 100 ng/ml sRANKL for 5 and 10 min. The whole cell lysates were subjected to Western blotting to determine phosphorylated or whole mTOR (*C* and *D*) or p38 (*E* and *F*). *C* and *E*, the representative blotting images from three independent experiments with similar results are shown. The entire gel and all duplicate images are placed in Figs. S5 and S6. *D* and *F*, upon quantification of each blot band, the ratios of phospho-mTOR/whole-mTOR (pmTOR/mTOR) (*D*) and phospho-p38/whole-p38 (pp38/p38) (*F*) are shown. *G*, control or TAS1R3 cells were treated with 100 ng/ml sRANKL with 0.05 μM SB202190 for 4 days. Representative images of osteoclasts visualized by TRAP staining from three independent experiments with similar results are shown. Scale bar represents 200 μm. *H*, the number of TRAP-positive cells with three or more nuclei (total) and 30 or more nuclei (cells with ≥30 nuclei) were counted. *I*, the expression levels of *Acp5 or Ctsk* were determined by quantitative PCR. *B*, *D*, *F*, *H*, and *I*, the bar graphs (mean ± SD) with dot plots presenting each sample from three or four independent experiments are shown (∗*p* < 0.05). mTOR, mammalian target of rapamycin; RANKL, receptor activator of nuclear factor-kappa B ligand; TAS1R, taste receptor type 1; TRAP, tartrate-resistant acid phosphatase.

**Figure 6:**
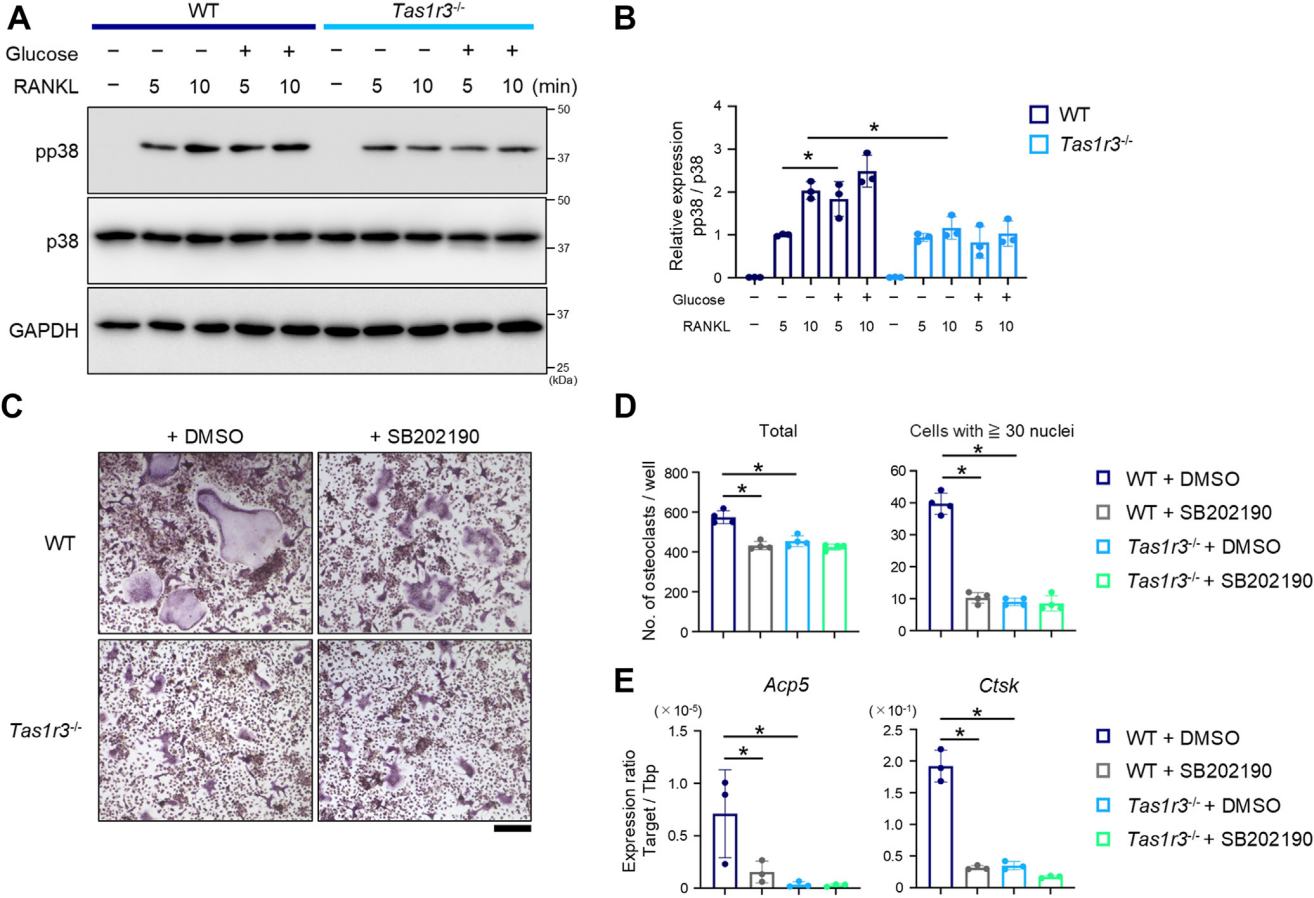
**Glucose–TAS1R3 axis promotes osteoclast differentiation by changing phosphorylated status of p38 in primary osteoclast.***A* and *B*, bone marrow cells from WT and *Tas1r3*^−/−^ mice were cultured in the presence of 100 ng/ml sRANKL and 30 ng/ml M-CSF with or without 5 mM glucose for 5 and 10 min. The whole cell lysates were subjected to Western blotting to determine phosphorylated or whole p38. *A*, the representative blotting images from three independent experiments with similar results are shown. The entire gel and all duplicate images are placed in Fig. S7. *B*, upon quantification of each blot band, the ratios of phospho-p38/whole-p38 (pp38/p38) are shown. *C* and *D*, bone marrow cells from WT and *Tas1r3*^−/−^ mice were cultured in the presence of 100 ng/ml sRANKL and 30 ng/ml M-CSF with or without 1 μM SB202190 for 6 days. *C*, representative images of osteoclasts visualized by TRAP staining from three independent experiments with similar results are shown. Scale bar represents 200 μm. *D*, the number of TRAP-positive cells with three or more nuclei (total) and 30 or more nuclei (cells with ≥30 nuclei) was counted (*D*). *E*, the expression levels of *Acp5 or Ctsk* were determined by quantitative PCR. *B*, *D*, *E*, the bar graph (mean ± SD) with dot plots presenting each sample from three or four independent experiments is shown (∗*p* < 0.05). M-CSF, macrophage colony-stimulating factor; RANKL, receptor activator of nuclear factor-kappa B ligand; TAS1R, taste receptor type 1; TRAP, tartrate-resistant acid phosphatase.

To elucidate the mechanism by which TAS1R3 signaling promotes osteoclast differentiation, we examined the phosphorylation levels of downstream effectors stimulated by RANK–RANKL signaling. In TAS1R3 cells, p38 phosphorylation was elevated compared with control cells 5 and 10 min after RANKL stimulation. In addition, phosphorylated p38 expression was further increased in TAS1R3 cells after 5 and 10 min of combined RANKL and glucose stimulation, compared with that in cells not stimulated with glucose. However, the levels of phosphorylated p65 or extracellular signal–regulated kinase were almost comparable in control and TAS1R3 cells under unstimulated and stimulated conditions (Fig. 5, *A* and *B*). Furthermore, TAS1R3 overexpression and glucose stimulation did not affect the protein levels of phosphorylated mammalian target of rapamycin (mTOR) (Fig. 5, *C* and *D*) despite TAS1R3's known regulation of mTOR signaling in skeletal muscle, heart, or pancreatic β cells (22). To further understand the role of the increased pp38 protein levels mediated by the glucose–TAS1R3 axis in osteoclast differentiation, we used SB202190, a p38 kinase inhibitor. Treatment with SB202190 strongly inhibited the increase in pp38 protein induced by the glucose–TAS1R3 pathway (Fig. 5, *E* and *F*). Consistently, TAS1R3-mediated increase in the number of TRAP-positive osteoclasts but not its basal level was significantly suppressed by SB202190 (Fig. 5, *G* and *H*). Although the basal levels of *Acp5* and *Ctsk* expression were slightly inhibited (43% and 40%, respectively), their enhanced expression in TAS1R3-overexpressing cells was more severely suppressed (51% and 78%, respectively) by SB202190 (Fig. 5*I*). Finally, we confirmed the role of TAS1R3 in osteoclast differentiation using BMMs from *Tas1r3*^−/−^ mice. Consistent with the findings in RAW 264.7 cells, RANKL-induced increase in the level of pp38 was further upregulated by glucose treatment in WT mouse BMMs. The RANKL-induced p38 phosphorylation and its augmentation by glucose treatment were alleviated in *Tas1r3*^−/−^ cells (Fig. 6, *A* and *B*). The number of TRAP-positive osteoclasts derived from WT BMMs, particularly those with 30 or more nuclei, was significantly reduced by SB202190 treatment. The osteoclastogenesis (Fig. 6, *C* and *D*) and the related gene expression (Fig. 6*E*) were downregulated in BMMs from *Tas1r3*^−/−^ mice. Consistent with the lack of influence on osteoclastogenesis of BMMs from *Tas1r3*^−/−^ mice by SB202190 (Fig. 6, *C* and *D*), the suppressive effect of SB202190 on *Acp5* and *Ctsk* expression in WT cells (78% and 84%, respectively) was stronger than that in TAS1R3-deficient cells (21% and 51%, respectively) (Fig. 6*E*). These data suggested that the glucose–TAS1R3 axis promotes osteoclastogenesis, at least in part, *via* increased phosphorylation of p38.

## Discussion

In this study, we demonstrated that TAS1R3 was expressed in osteoclasts and regulates osteoclastogenesis. Furthermore, the TAS1R3 homodimer can detect glucose, and this signaling promotes osteoclast differentiation by enhancing the phosphorylation status of p38.

TAS1R3 global knockout (18) and loss-of-function TAS1R3 mutant mice (19) showed high bone mass, particularly when fed an HFS diet. However, the cell types that express TAS1R3 and their relationship with bone phenotypes remain unclear. Typically, high bone mass results from a relative increase in bone formation compared with bone resorption. However, it is uncertain whether bone formation increases, bone resorption decreases, or a combination of both occurs under TAS1R3-knockout conditions. In this study, we showed that TAS1R3 is highly expressed in osteoclasts but barely expressed in osteoblasts. In addition, osteoclast differentiation decreased in the absence of TAS1R3, strongly suggesting that the high bone mass phenotype observed in TAS1R3 dysfunction was due to reduced bone resorption by osteoclasts. Furthermore, the overexpression of TAS1R3 in combination with glucose or cyclamate enhanced osteoclast differentiation. This may also explain the pronounced bone phenotype observed in TAS1R3 global knockout mice fed with an HFS diet (18, 19).

Functional expression of the sweet taste receptors, TAS1R2–TAS1R3, has been reported in several metabolic tissues (7). TAS1R2 conventional knockout mice also showed a high bone mass phenotype, especially when fed an HFS diet (18). These phenotypes suggest that the mechanism, not *via* direct action on osteoclasts, including systemic metabolic alteration, affects bone mass in the absence of sweet receptors because TAS1R2 has almost no expression in osteoclasts (Fig. 2*A*). Thus, the bone mass phenotype in loss-of-function TAS1R3 mutant mice may also be affected by direct osteoclast function as well as by other mechanisms such as systemic metabolic changes. Further experiments using osteoclast lineage–specific knockout mice are needed to better understand the function of TAS1R3 in osteoclasts. Recently, the crucial contribution of nucleotide-binding domain, leucine-rich–containing family, pyrin domain–containing-3 inflammasome in high-fat diet–induced type 2 diabetes and resulting bone loss has been reported (23), indicating the requirement of further investigation into the relationship between the nucleotide-binding domain, leucine-rich–containing family, pyrin domain–containing-3 inflammasome and TAS1R3.

In the taste buds, TAS1R3 heterodimerizes with TAS1R1 or TAS1R2 to form functional taste receptors (2). However, in our study, TAS1R3 detected glucose or cyclamate and induced signal transduction without TAS1R1 or TAS1R2. TAS1R family proteins usually function by forming dimers (2). A recent study reported that TAS1R3 functions as a homodimer in pancreatic β cells (24). Our *in silico* experiments also indicated that TAS1R3 has sufficient potential to form homodimers. Therefore, we predicted that TAS1R3 in osteoclasts, as in pancreatic β cells, is likely to form and function as a homodimer.

Osteoclasts have a high demand for energy sources during active bone resorption as they move around the bone surface (16, 17). Metabolites generated from aerobic respiration in the mitochondria regulate osteoclast differentiation *via* DNA methylation (17). The uptake of carbohydrates into the cell and utilization of energy and metabolites produced as cellular components are fundamental processes common to all cells. In the intestine, the TAS1R2–TAS1R3 complex senses sugars and induces the expression of the sodium-dependent glucose transporter isoform 1 (SGLT1), which promotes sugar uptake into cells (25). This suggests that TAS1R3 is involved in metabolic regulation by promoting sugar uptake and influencing osteoclast differentiation. However, we did not observe an increase in SGLT1 mRNA levels in TAS1R3 cells compared with the controls (data not shown). In addition, treatment with sodium cyclamate, which does not participate in energy metabolism, increased osteoclast differentiation in TAS1R3-expressing cells. These results indicate that TAS1R3 has an energy metabolism–independent regulatory mechanism for osteoclast differentiation.

In this study, we demonstrated that TAS1R3 upregulates the p38 signaling pathway when stimulated by glucose. In the original function of taste receptors in taste buds, elevated intracellular Ca^2+^ activates TRPM5, causing membrane depolarization, the generation of action potentials, that results in release of ATP, and then activation of afferent nerve fibers to induce taste sensation (2). Regarding intracellular signaling, the TAS1R1–TAS1R3 complex responds to amino acids and enhances mTOR signaling (22). However, mTOR signaling was not involved in the function of TAS1R3 in osteoclastogenesis in the present study (Fig. 5, *C* and *D*). Instead, TAS1R3, which is expressed in osteoclasts, enhances p38 signaling. As p38 is essential for osteoclast differentiation, and its signal intensity correlates with the degree of osteoclast differentiation (11, 26), we hypothesized that the enhancement of p38 signaling by activation of TAS1R3 may promote osteoclast differentiation. These results suggest that the signaling pathway differs between TAS1R3 alone and the TAS1R1–TAS1R3 complex. Based on our findings, we expect that the significance of sugar and amino acid reception in tissues with high TAS1R3 expression, such as the kidneys and muscles, will be better understood.

GPCRs are of great interest as pharmacological targets because they regulate a wide variety of physiological processes and have cell surface access sites suitable for drug discovery. It is estimated that 35% of marketed drugs act directly on GPCRs (27). In this study, we found that TAS1R3 promoted osteoclast differentiation in response to glucose or cyclamate without TAS1R1 or TAS1R2. In humans, patients with obesity and diabetes have an increased risk of periodontal diseases and bone fractures (28, 29, 30, 31). Our findings indicate that glucose reception by TAS1R3 in osteoclasts enhances osteoclastogenesis, which may contribute to the development of these bone metabolic diseases. Therefore, targeting TAS1R3 in osteoclasts could offer a potential therapeutic approach for treating bone metabolic diseases. Lactizole and gymnemic acid have been reported as human TAS1R3 inhibitors (32, 33). These agents are promising candidates for the treatment of bone metabolic diseases. TAS1R3 functions as a receptor by forming a heterodimer with TAS1R1 or TAS1R2 in several tissues, but TAS1R3 could form a homodimer and function as a glucose sensor in case of osteoclasts. Therefore, pharmacological agents targeting TAS1R3 homodimer could be potential candidates for the treatment of metabolic bone diseases with fewer side effects.

Recently, remarkable progress has been made in the development of drug delivery systems for bone tissue (34, 35), materials for bone and cartilage regeneration (36, 37), and materials for diabetic wound regeneration (38). The ability to locally regulate the glucose–TAS1R3 axis in the bone using these latest technologies has the potential to contribute to human health.

The potential limitations of this study should be considered when interpreting its results. First, TAS1R3 was not completely absent in the osteoblast lineage cells. Therefore, to fully understand the role of TAS1R3 in bone metabolism, it may be necessary to conduct experiments that elucidate the relationship between osteoclasts and osteoblast lineage cells, particularly in terms of TAS1R3’s contribution. Second, glucose is sensed by the TAS1R2–TAS1R3 heterodimer, and TAS1R1–TAS1R3 detects specific amino acids and nucleic acids. It would be beneficial to evaluate whether amino acids and/or nucleic acids regulate osteoclast differentiation by interacting with the TAS1R3 homodimer. An *in vivo* bone metabolism study using conditional knockout mice in which TAS1R3 is specifically absent in osteoclasts is useful for clarifying the biological significance of this extraordinarily expressed TAS1R3.

## Experimental procedures

### Animals and preparation of tissues and cells

*Tas1r3* conventional knockout mice were kindly provided by Dr Robert F Margolskee of the Monell Chemical Senses Center (39). After euthanasia, various tissues and cells were collected to examine the mRNA expression. Mouse femurs were collected, both ends of the bone tissue were removed with forceps, and the bone marrow was flushed with a 24G needle and 10 ml syringe to obtain BMCs for osteoclast differentiation experiments. All mice were handled in accordance with the Animal Care and Use Committee of Kyushu Dental University, based on the Animal Research: Reporting of *In Vivo* Experiments (ARRIVE) guidelines (approval number: 22-008).

### Cell culture and osteoclast and osteoblast differentiation

Bone marrow cells isolated from 4- to 6- and 13-week-old mice were cultured in MEMα with l-glutamine and Phenol Red (MEMα) (Fujifilm Wako) supplemented with 10% (v/v) fetal bovine serum (FBS) (Sigma–Aldrich) and 1% (v/v) penicillin–streptomycin (Thermo Fisher Scientific). After incubation for 3 h, nonadherent cells were collected and cultured with 100 ng/μl macrophage colony-stimulating factor (Fujifilm Wako) for 3 days to differentiate into BMMs (40). RAW 264.7 cells (Research Resource Identifier: CVCL_0493) and human embryonic kidney 293 cells (Research Resource Identifier: CVCL_0045) were obtained from KAC Co, Ltd and RIKEN Cell Bank, respectively. Both cell lines were cultured in Dulbecco's modified Eagle’s medium (Fujifilm Wako) supplemented with 10% (v/v) FBS and 1% (v/v) penicillin–streptomycin (40). To induce osteoclastogenesis, BMMs were spread on a plastic dish at 1.0 × 10^6^ cells/cm^2^. Cells were treated with αMEM supplemented with 10% FBS, 30 ng/ml macrophage colony-stimulating factor, and 90 or 100 ng/ml sRANKL (Oriental Yeast) for 7 days. RAW 264.7 cells were spread on a plastic dish at 1.0 × 10^4^ cells/cm^2^ density in αMEM supplemented with 10% FBS and 50 or 100 ng/ml sRANKL for 4 days. The culture medium was changed every 2 days. To examine the ligand, cells were treated with 5 mM glucose (Fujifilm Wako) or 1 μM cyclamate (Tokyo Chemical Industry) for 4 days. To stop p38 signaling, we used the p38 inhibitor SB202190 (Selleck). Cells were treated with or without 0.05 to 1 μM SB202190 for 1 h or 4 days. Primary osteoblasts were prepared from neonatal murine calvarial bone as described previously (41). Briefly, after treatment with 1 mg/ml collagenase (Fujifilm Wako) and 2 mg/ml dispase (Thermo Fisher Scientific), the bone cells were cultured for 7 days in MEMα containing 10% FBS, 1% penicillin–streptomycin, 50 μg/ml ascorbic acid (Fujifilm Wako), and 10 mM β-glycerophosphate (Merck).

### Generating RAW 264.7 cells stably expressing TAS1R3

Mouse *Tas1r3* complementary DNA (cDNA) was subcloned from a mouse osteoclast cDNA library by standard PCR and inserted into the pcDNA3.1/V5-His TOPO vector (Thermo Fisher Scientific). Plasmids were introduced into RAW 264.7 cells, using Lipofectamine 2000 (Thermo Fisher Scientific) according to the manufacturer’s protocol. Cells constitutively expressing *Tas1r3* were selected *via* treatment with 500 μg/ml geneticin (G418) for 14 days (Fujifilm Wako) (42).

### TRAP and DAPI staining and evaluation of osteoclastogenesis

Cells were fixed with 10% formaldehyde for 10 min, followed by an ethanol–acetone (1:1) mixture for 1 min, and washed with distilled water. The fixed cells were stained using a leukocyte acid phosphatase (TRAP) staining kit (Sigma–Aldrich) according to the manufacturer's protocol. The resulting preparation was observed under light microscopy, then washed with distilled water, and stained with 4′,6-diamidino-2-phenylindole (DAPI; Dojindo) diluted 1:100 for 20 min. Overlapping TRAP- and DAPI-staining images were used to count the nuclear number in the cells (Fig. S8). TRAP-positive multinuclear cells (≥3 nuclei) were regarded as osteoclasts (40), and osteoclasts with 30 or more nuclei are considered mature osteoclasts (43, 44, 45). Quantification was performed independently by two researchers using a microscope.

### RNA extraction, cDNA synthesis, and real-time PCR

Total RNA from each tissue and cell was isolated using the FastGene RNA Basic Kit (Nippon Genetics) according to the manufacturer’s instructions. cDNA was synthesized from 1 μg of total RNA using a High-Capacity cDNA Reverse Transcription Kit (Thermo Fisher Scientific). Real-time PCR was performed using PowerUp SYBR Green Master Mix (Thermo Fisher Scientific) and a QuantStudio 3 Real-time PCR system (Thermo Fisher Scientific). Primers used are listed in Table 1.

**Table 1:** Quantitative PCR primers

Gene	Forward primer (5′ to 3′)	Reverse primer (5′ to 3′)
*Acp5*	Tcctggctcaaaaagcagtt	Acatagcccacaccgttctc
*Ctsk*	Gggaagcaagcactggataa	ccgagccaagagagcatatc
*Atp6v0a3*	ctcatcaggaccaaccgcttca	cgccaaacatcacagcgaagag
*Nfatc1*	ggtgctgtctggccataact	gcggaaaggtggtatctcaa
*Tbp*	gaagctgcggtacaattccag	ccccttgtacccttcaccaat
*Tas1r1*	actgctgcttcgagtgcat	acaaggctggcaggtgtg
*Tas1r2*	gtacacccccaacaacacg	gtggaggcctatgggttttt
*Tas1r3*	aacagcatcccgtgcaac	ccacagccatcttcatagcc

*Tbp*, *Ctsk*, *Acp5*, *Atp6v0a3*, and *Nfatc1* cDNA were amplified from the osteoclast cDNA library by RT–PCR and inserted into pcDNA3.1+ vector (Thermo Fisher Scientific). The cloning method of murine *Tas1r1*, *Tas1r2*, and *Tas1r3* cDNAs was described in the “*HiBit technology assay*” and “*Generating RAW 264.7 cells stably expressing TAS1R3*” sections. Each cDNA was stepwise diluted to 1 fg, 10 fg, 100 fg, 1 pg, and 10 pg to obtain the standard concentration–Ct curve. Referring to the standard curve, the amount of mRNA in each sample was determined from each Ct value (46). The resulting mRNA amount was normalized by the amount of *Tbp* simultaneously measured in the same real-time PCR plate and was expressed as the expression ratio between the sample and *Tbp*.

### Calcium imaging

RAW 264.7 cells were seeded (1.0 × 10^4^ cells/cm^2^) in glass-bottomed dishes (Matsunami Glass) and cultured overnight. The cells were washed with PBS and incubated in recording medium (20 mM Hepes, 115 mM NaCl, 5.4 mM KCl, 0.8 mM MgCl_2_, 1.8 mM CaCl_2_, and 5.6 mM glucose) supplemented with Fura2-AM (Dojindo), PluronicF-127 (Biotium), and 4-(dipropylsulfamoyl) benzoic acid (Combi-Blocks) at 37 °C for 1 h. Fura-2AM-loaded RAW 264.7 cells were perfused with 4-(dipropylsulfamoyl) benzoic acid in recording buffer for 5 min at room temperature. They were then perfused with 25 mM glucose or 10 μM cyclamate for 10 min. Using IX71 (Olympus) microscope and HC Image software (Hamamatsu Photonics), the fluorescence intensity of the cells was measured at excitation wavelengths of 340 and 380 nm, with results shown as 340/380 nm.

### Collection of protein and Western blotting and IP

Cells were lysed using radioimmunoprecipitation assay (RIPA) buffer (1% Triton X-100, 0.1% SDS, 1% sodium deoxycholate, 150 mM NaCl, 10 mM Tris, and 5 mM EDTA) containing Protease Inhibitor Cocktail Set I (Fujifilm Wako) and phosphatase inhibitor PhosSTOP (Merck). After sonication and centrifugation at 14,000*g* for 15 min at 4 °C, the supernatant was collected and boiled in sample buffer (125 mM Tris, 40% glycerol, 4% SDS, 0.2 M dithiothreitol, and 0.01% bromophenol blue) for 5 min at 95 °C. For IP, cells were lysed using RIPA buffer containing leupeptin (Fujifilm Wako) and PMSF (Fujifilm Wako). After centrifugation at 12,000 rpm for 15 min at 4 °C, the supernatant was collected. Next, 10 μl of DYKDDDDK Fab-Trap Agarose (Proteintech) was added and incubated at 4 °C for 1 h. After multiple washes with RIPA buffer, the samples were denatured with sample buffer for 5 min at 95 °C. The samples were separated using 10% SDS-PAGE gels and transferred to polyvinylidene fluoride membranes. The membranes were blocked with block ace (KAC Co, Ltd) for 1 h at room temperature and incubated overnight at 4 °C with corresponding primary antibody (Table 2) in block ace. The specificity of each antibody was confirmed by reviewing the manufacturer's instruction. The membranes were then washed with Tris-buffered saline with Tween-20 (2.5% Tween-20, 50 mM Tris, 150 mM NaCl, 65 mM KCl, adjust to pH 7.4 with HCl) and incubated with horseradish peroxidase–conjugated anti-mouse or anti-rabbit IgG secondary antibodies (Jackson ImmunoResearch Laboratories, Inc) in 5% bovine serum albumin and Tris-buffered saline with Tween-20. Finally, the blots were imaged using Immobilon ECL Ultra Western HRP Substrate (Merck) and ChemiDoc Touch MP (Bio-Rad). The expression level of each protein was quantified by using Image Lab 6.1 software (Bio-Rad).

**Table 2:** Primary antibodies

Primary antibody	Company and catalog no.:	Research Resource Identifier
Anti-p38 MAPK	Cell Signaling Technology #8690	AB_10999090
Anti-phospho-p38 MAPK	Cell Signaling Technology #9211	AB_331641
Anti-p44/42 MAPK (ERK1/2)	Cell Signaling Technology #9102	AB_330744
Anti-phospho-p44/42MAPK (ERK1/2)	Cell Signaling Technology #9101	AB_331646
Anti-NF kappaB p65	Cell Signaling Technology #8242	AB_10859369
Anti-phospho-NF kappaB p65	Cell Signaling Technology #3031	AB_330559
Anti-Mtor	Cell Signaling Technology #2972	AB_330978
Anti-phospho-mTOR	Cell Signaling Technology #2971	AB_330970
Anti-β-actin	Medical and Biological Laboratories Co Ltd #M177-3	AB_10697039
Anti-V5-tag	Medical and Biological Laboratories Co Ltd #PM003	AB_592941
Anti-DDDDK-tag	Medical and Biological Laboratories Co Ltd #M185-3L	AB_11123930

MAPK, mitogen-activated protein kinase.

### HiBit technology assay

Mouse *Tas1r1* and *Tas1r2* were subcloned from a murine tongue cDNA library into the pBiT2.1-C [TK/SmBiT] vector (Promega) (TAS1R1-SmBiT and TAS1R2-SmBiT). *Tas1r3* cDNA of the mTas1r3 V5-His vector was subcloned into pBiT1.1-C [TK/LgBiT] (Promega) (TAS1R3-LgBiT) and pBiT2.1-C [TK/SmBiT] (Promega) (TAS1R3-SmBiT). These vectors and pGL4.53 [luc2/PGK] (Promega) were cotransfected into human embryonic kidney 293 cells using Lipofectamine 2000. After 24 h, ONE-Glo EX luciferase assay reagent (Promega) was added, and firefly luciferase activity was measured using an Infinite 200 plate reader (Tecan Japan). NanoDLR Stop & Glo reagent (Promega) was added, and NanoLuc luminescence was measured using the Infinite 200 plate reader. NanoLuc luminescence was normalized to firefly luciferase activity.

### Structure analysis

The amino acid sequence of mouse TAS1R3 (1–356) was uploaded to Colaboratory AlphaFold2 (ColabFold v1.5.5: AlphaFold2 using MMseqs2; DeepMind Technologies Limited), and the default settings were used for all runs to predict the homodimer structure of the protein (47, 48).

### Data analysis and statistics

Experiments were conducted independently at least three times, and data are presented as mean ± SD. Statistical significance between groups was analyzed by Student's *t* test for comparisons between two groups or one-way ANOVA and Tukey–Kramer *post hoc* test for comparisons among three or more groups. Results were considered statistically significant at *p* < 0.05.

## Data availability

Original images and Western blot images were deposited in Mendeley and are publicly available on the date of publication. The DOI is 10.17632/zj8w74j2rr.1. All data reported in this article will be shared with the lead contact upon request.

## Supporting information

This article contains supporting information (Supplemental Figs. S1–S8).

## Conflict of interest

The authors declare that they have no conflicts of interest with the contents of this article.
