# Supplementary material for: Taste receptor type 1 member 3 in osteoclasts regulates osteoclastogenesis via detection of glucose
Source: J Biol Chem. 2025 Feb 6;301(3):108273. doi: 10.1016/j.jbc.2025.108273 (PMC11925095; doi:10.1016/j.jbc.2025.108273)
Supplement: Supporting information [file mmc1.pdf]

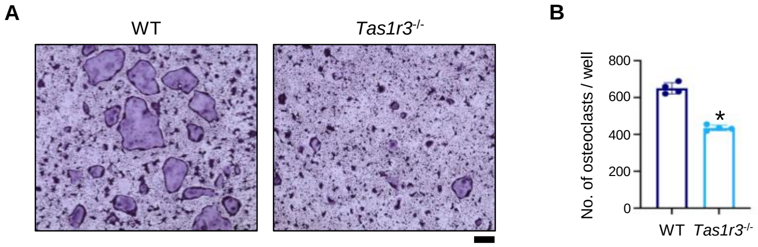

Supplemental figure S1. TAS1R3 plays a pivotal role in osteoclast differentiation even in old mice
(A, B) Osteoclasts were differentiated from bone marrow cells collected from 13-week-old wild-type (WT) or *Tas1r3* conventional knockout (*Tas1r3*$^{-/-}$) mice. Osteoclastogenesis was stimulated by 30 ng/ml M-CSF and 100 ng/ml sRANKL. Osteoclasts were visualized by TRAP staining (A), and the number of TRAP-positive cells with 3 or more nuclei was counted (B). Representative images of three independent experiments with similar results are shown. Scale bar = 200 μm (B). The bar graphs (mean ± SD) with dot plots presenting each sample from four independent experiments are shown (*; $p < 0.05$)

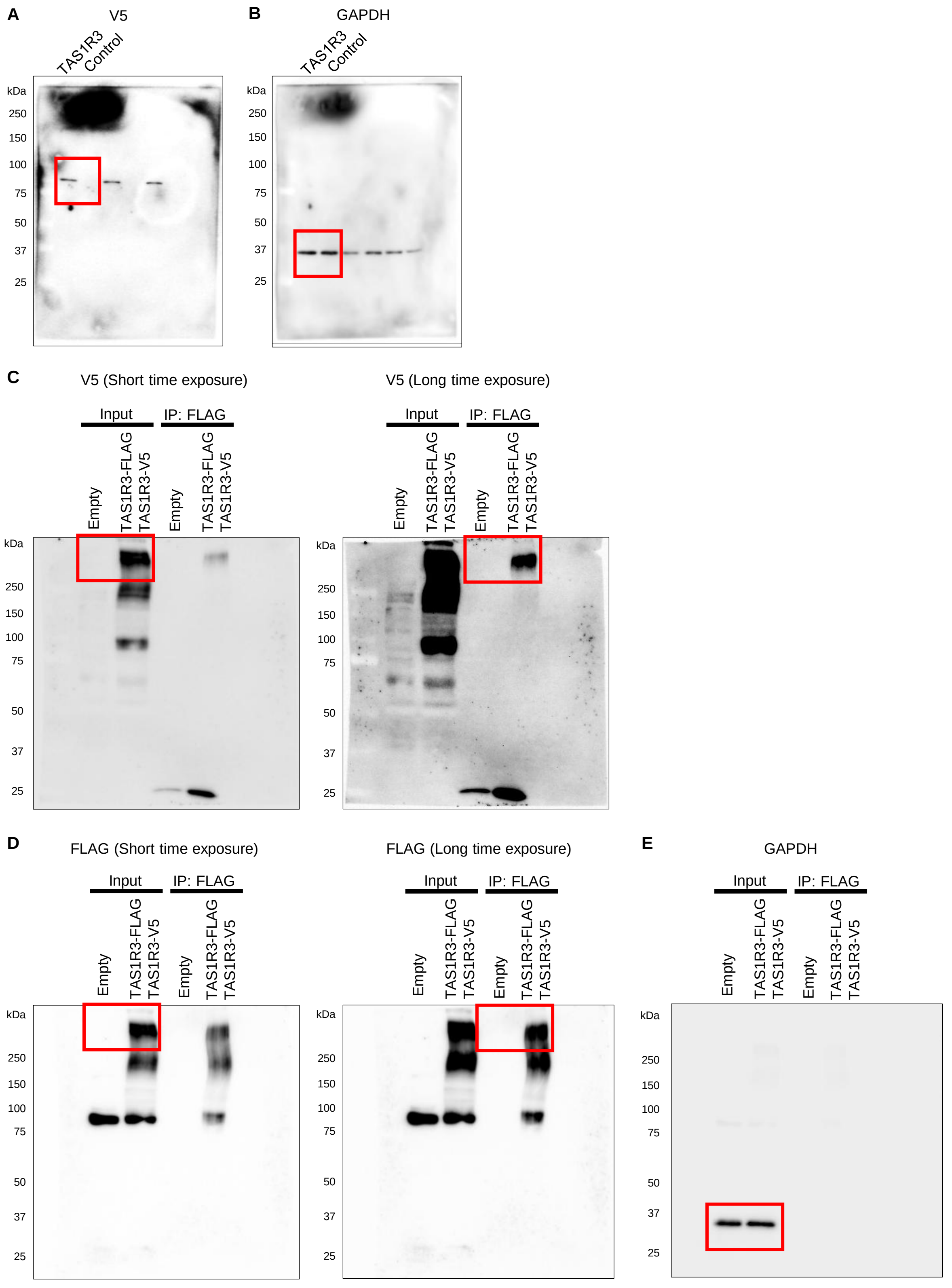

Supplemental figure S2. Full uncropped blots of Figure 2C and 2G.
(A, B) Uncropped image of V5 (A) or GAPDH (B) in Figure 2C.
(C-E) Uncropped image of V5 (C), FLAG (D) or GAPDH (E) in Figure 2G.

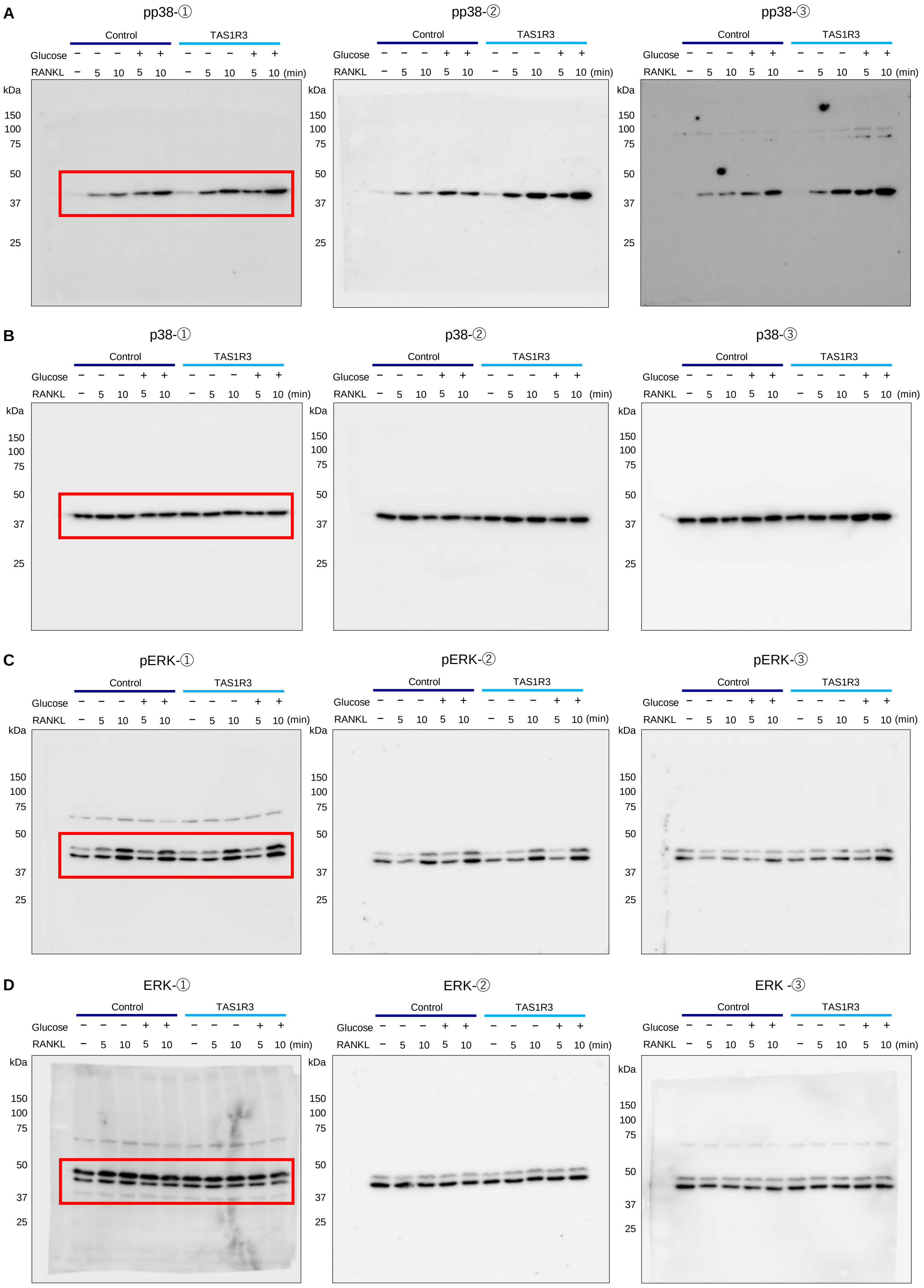

Supplemental figure S3. Full uncropped blots of pp38, p38, pERK and ERK in Figure 5A.
(A-D) Uncropped image of pp38 (A), p38 (B), pERK (C), ERK (D) in Figure 5A.

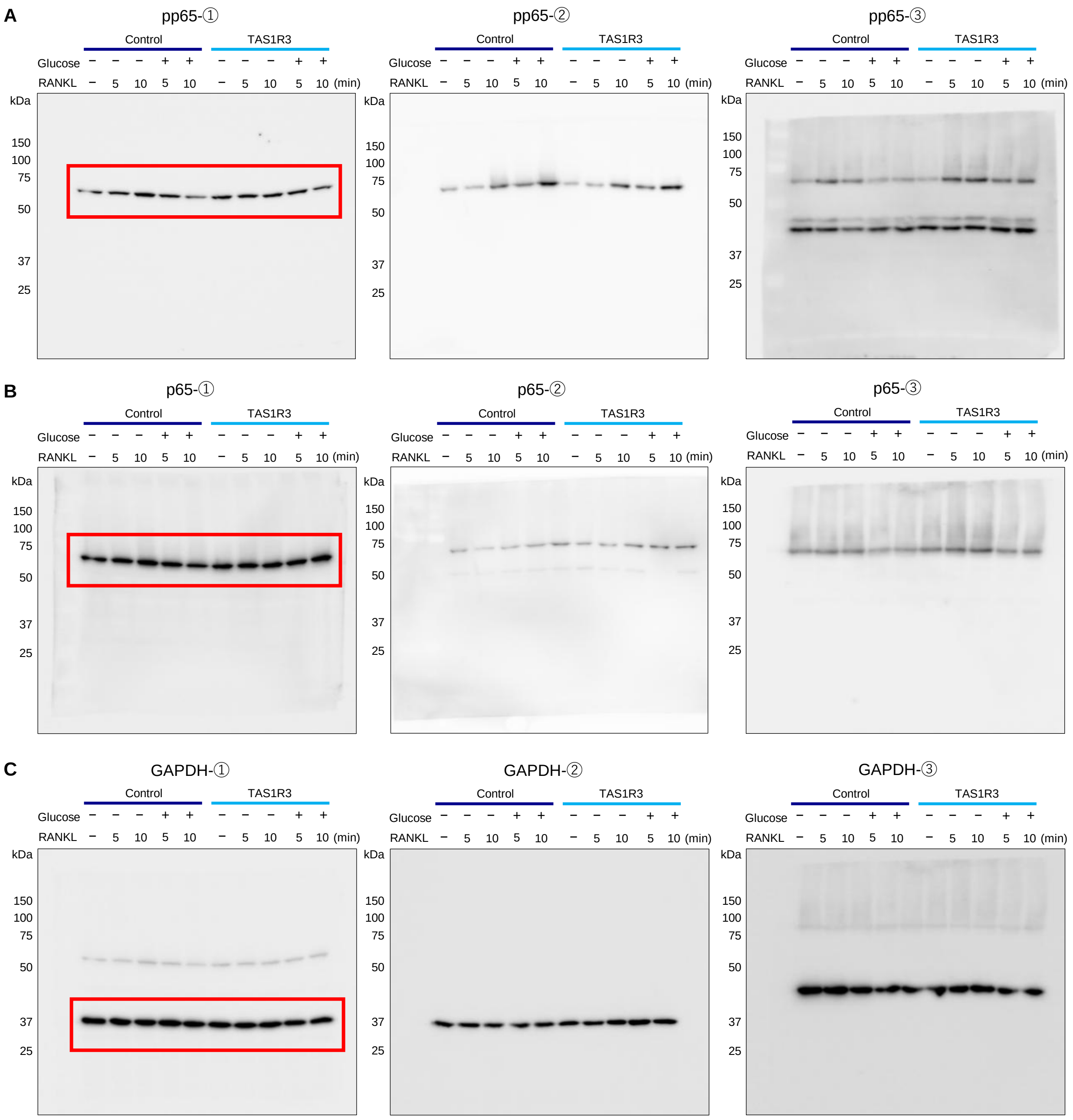

Supplemental figure S4. Full uncropped blots of pp65, p65 and GAPDH in Figure 5A.
(A-C) Uncropped image of pp65 (A), p65 (B) or GAPDH (C) in Figure 5A.

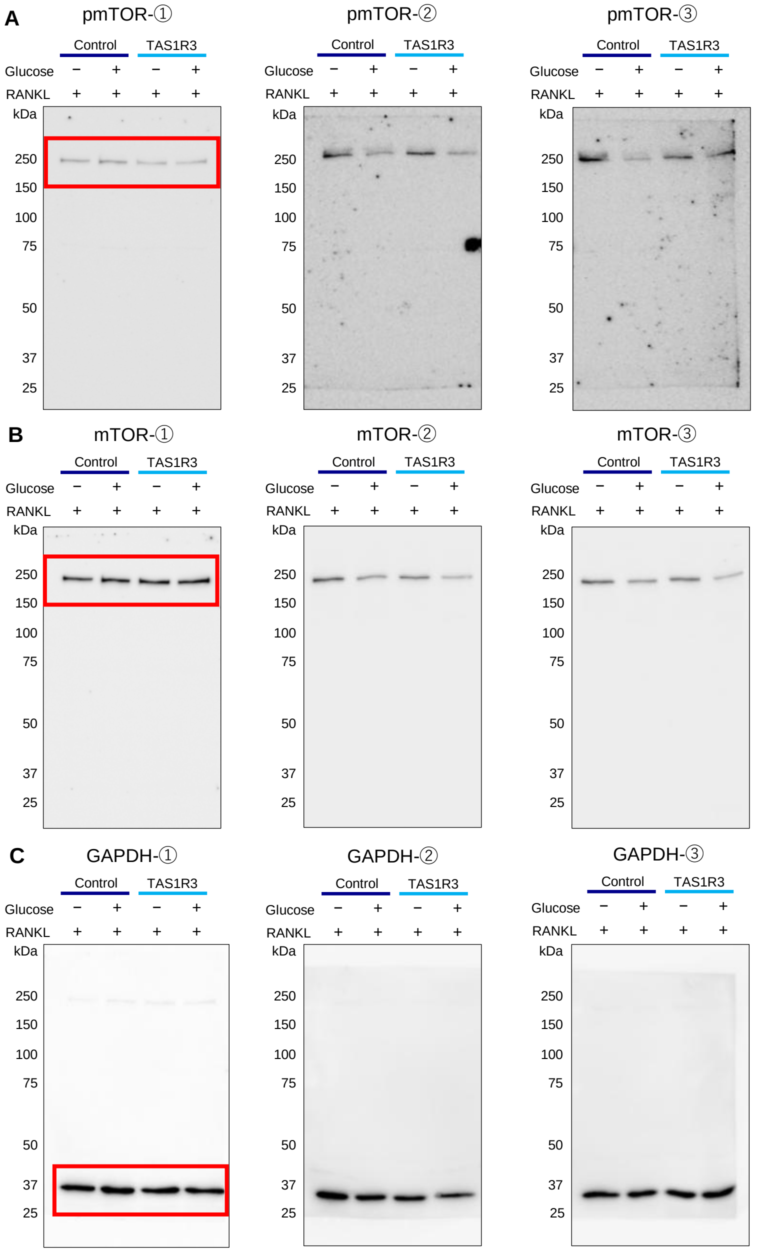

Supplemental figure S5. Full uncropped blots of Figure 5C.
(A-C) Uncropped image of pmTOR(A), mTOR(B) or GAPDH (C) in Figure 5C.

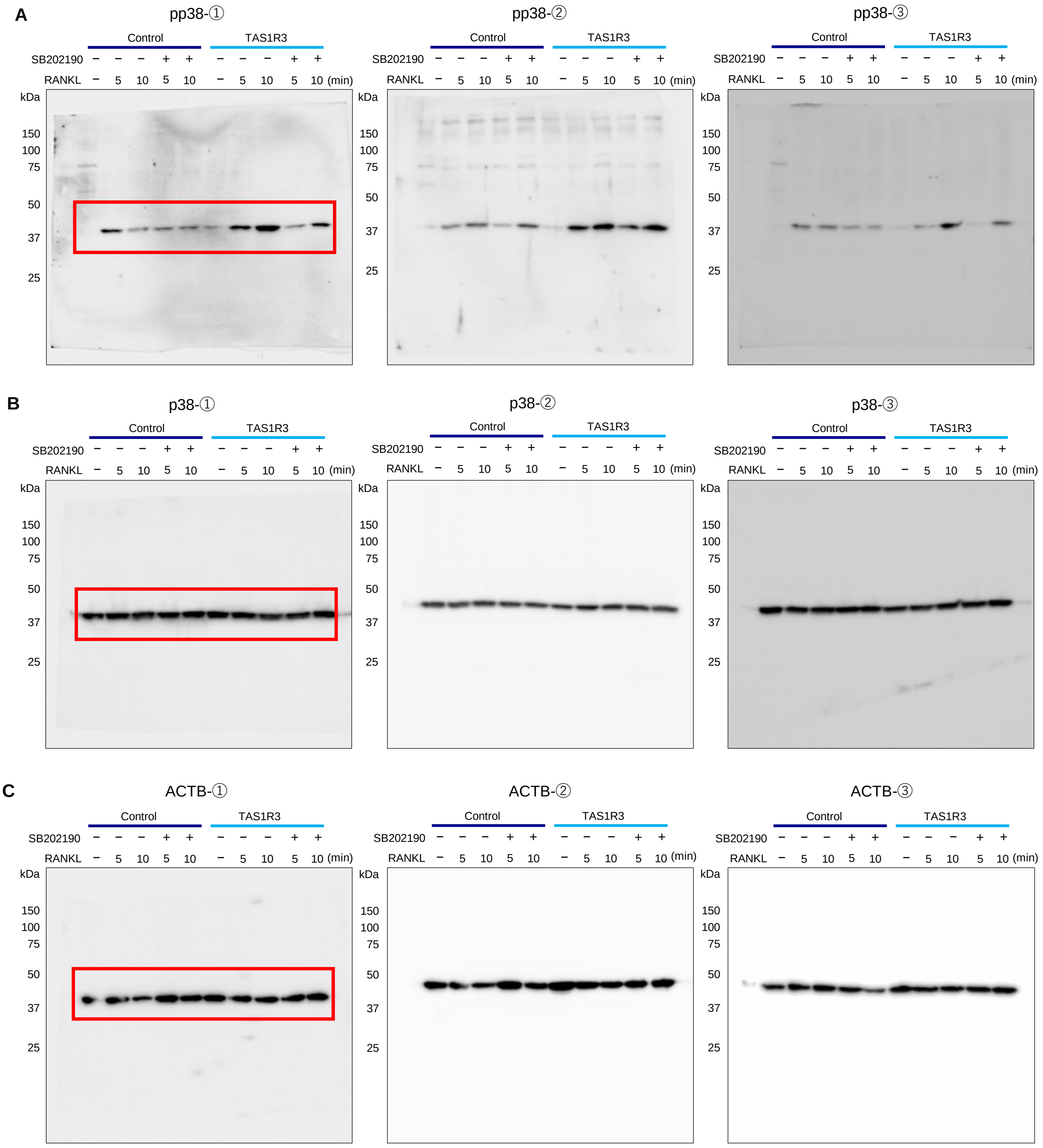

Supplemental figure S6. Full uncropped blots of Figure 5E.
(A-C) Uncropped image of pp38(A), p38(B) or ACTB(C) in Figure 5E.

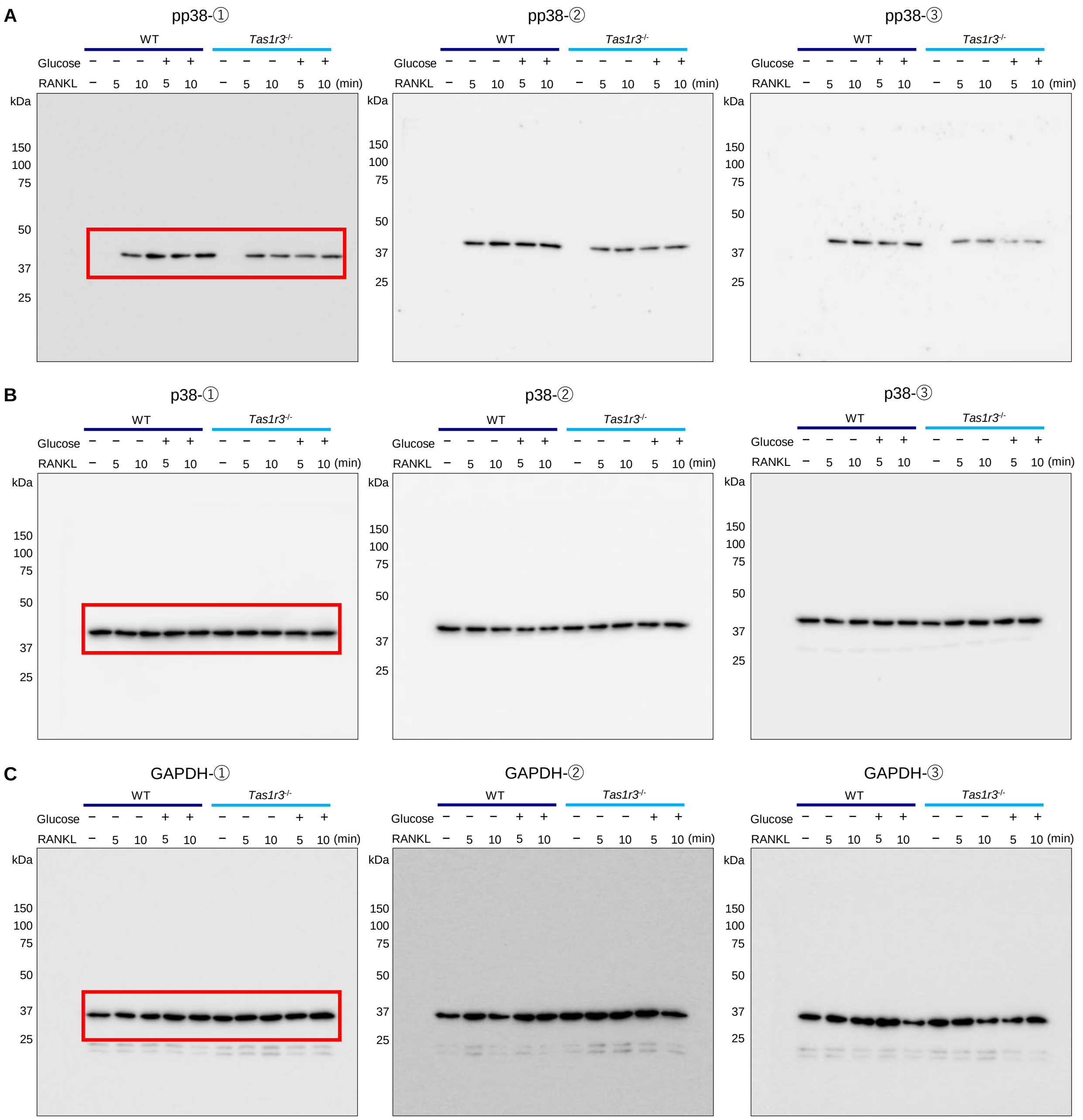

Supplemental figure S7. Full uncropped blots of Figure 6A.

(A-C) Uncropped image of pp38(A), p38(B) or GAPDH(C) in Figure 6A.

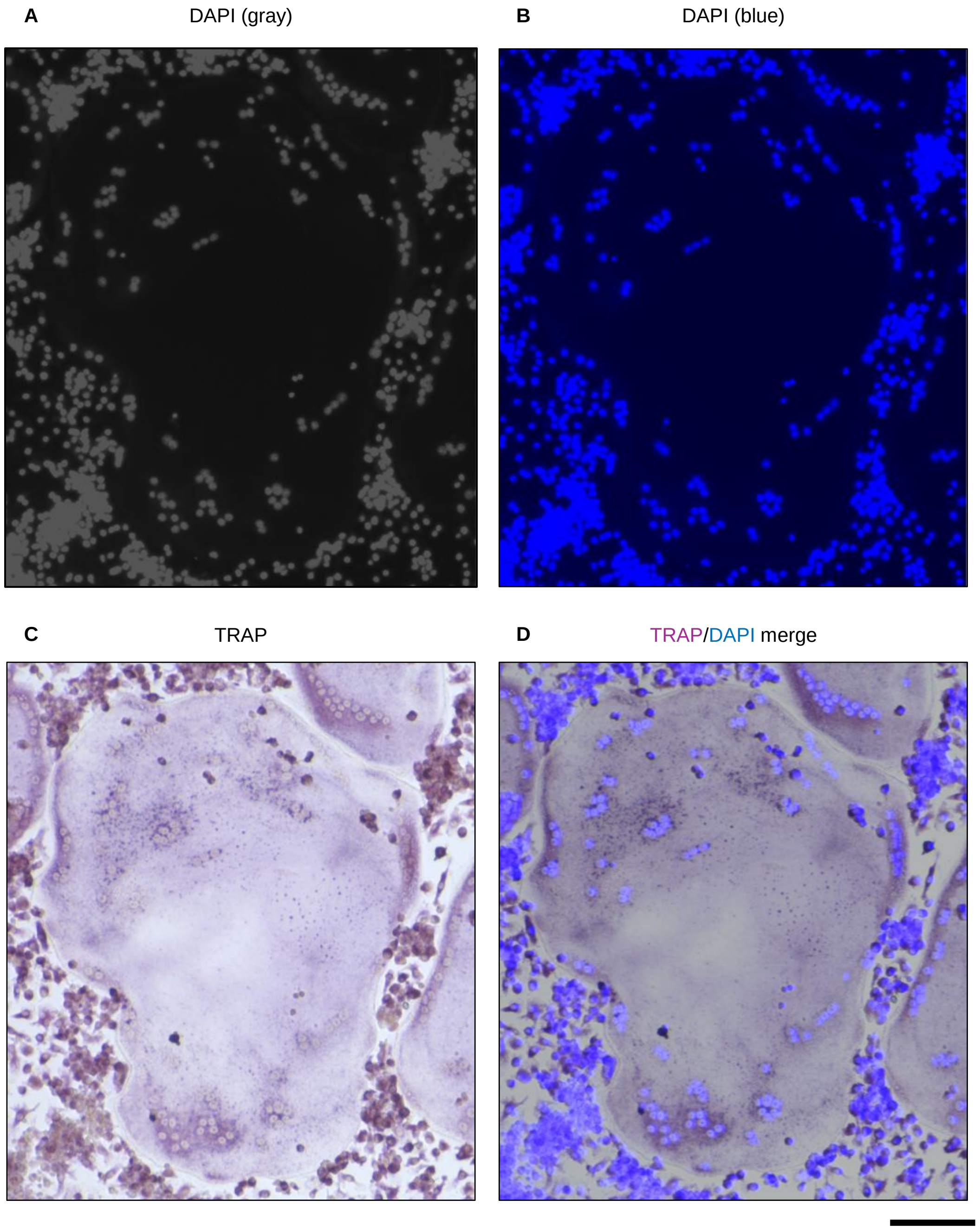

Supplemental figure S8. Overlapping TRAP- and DAPI-staining images to measure the number of nuclei

(A, B, C) Osteoclasts were visualized by DAPI-staining shown in gray (A), DAPI-staining shown in blue for merge image (B) and TRAP-staining shown in purple (C). (D) TRAP-staining and DAPI-staining images were merged. Scale bar = 100 µm
